# Subcellular Localization of Epstein–Barr Virus BLLF2 and Its Underlying Mechanisms

**DOI:** 10.3389/fmicb.2021.672192

**Published:** 2021-07-22

**Authors:** Jingjing Li, Yingjie Guo, Yangxi Deng, Li Hu, Bolin Li, Shenyu Deng, Jiayi Zhong, Li Xie, Shaoxuan Shi, Xuejun Hong, Xuelong Zheng, Mingsheng Cai, Meili Li

**Affiliations:** ^1^The Second Affiliated Hospital, State Key Laboratory of Respiratory Disease, Guangdong Provincial Key Laboratory of Allergy & Clinical Immunology, Guangzhou Medical University, Sino-French Hoffmann Institute, School of Basic Medical Science, Guangzhou Medical University, Guangzhou, China; ^2^Department of Oncology, Affiliated Hospital of Weifang Medical University, Weifang, China; ^3^Centralab, Shenzhen Center for Chronic Disease Control, Shenzhen, China

**Keywords:** EBV BLLF2, NLS, NES, CRM1, TAP, importin

## Abstract

Epstein–Barr virus (EBV), the pathogen of several human malignancies, encodes many proteins required to be transported into the nucleus for viral DNA reproduction and nucleocapsids assembly in the lytic replication cycle. Here, fluorescence microscope, mutation analysis, interspecies heterokaryon assays, co-immunoprecipitation assay, RNA interference, and Western blot were performed to explore the nuclear import mechanism of EBV encoded BLLF2 protein. BLLF2 was shown to be a nucleocytoplasmic shuttling protein neither by a chromosomal region maintenance 1 (CRM1)- nor by a transporter associated with antigen processing (TAP)-dependent pathway. Yet, BLLF2’s two functional nuclear localization signals (NLSs), NLS1 (^16^KRQALETVPHPQNRGR^31^) and NLS2 (^44^RRPRPPVAKRRRFPR^58^), were identified, whereas the predicted NES was nonfunctional. Finally, BLLF2 was proven to transport into the nucleus *via* a Ran-dependent and importin β1-dependent pathway. This mechanism may contribute to a more extensive insight into the assembly and synthesis of EBV virions in the nucleus, thus affording a new direction for the treatment of viruses.

## Introduction

Epstein–Barr virus (EBV), a member of the gamma herpes virus subfamily, is the most universal and persistent pathogen that prevails in humanity, with almost 90% of the world’s population maintaining a lifelong subclinical infection (Young et al., 2016). As the first discovered human tumor virus, EBV is related to several human malignancies, including infectious mononucleosis, nasopharyngeal carcinoma, Burkitt lymphoma, various lymphoproliferative disorders, and endemic Hodgkin lymphoma (Lieberman, 2014). Although EBV holds a latent infection in the host cells, it can intermittently exchange from the latent phase to lytic cycle, followed by the induction of more than 80 viral constituents, the synthesis of viral genomic DNA, and eventually the production of progeny virions (Hammerschmidt and Sugden, 2013). Upon stimulation, the viral immediate-early (IE) transcriptional activators Rta and Zta are first expressed to enhance early (E) genes, which include those essential for viral DNA genome replication. After viral DNA amplification in the replication focuses of the nucleus, viral late (L) gene transcription takes place; this process encodes diverse categories of viral structural components, such as capsid proteins, tegument proteins, and glycoproteins, which are vital for DNA replication, viral morphogenesis, or virion composition (Hammerschmidt and Sugden, 2013). Several EBV-encoded proteins are transported into the nucleus for viral DNA replication and nucleocapsid assembly in the lytic phase.

The trafficking of target proteins between the nucleus and cytoplasm of a eukaryote is achieved through the nuclear pore complex (NPC) embedded in the nuclear membrane, which bears an extremely preserved architecture with an eightfold rotational symmetry that carries a central aqueous cylindrical tunnel embraced with a huge number of specialized appendages (Belov et al., 2004). Dissimilar with a small molecule that moves into and out of the nucleus on the way of simple diffusion, the nucleocytoplasmic shuttling of a large molecule is accomplished *via* various cellular transporter exportins and importins by distinguishing favorable motifs on the target proteins named nuclear localization signal (NLS) and nuclear export signal (NES) (Belov et al., 2004).

Nuclear trafficking of a definitive protein is often achieved by the canonical importin α/β-reliant nuclear translocation pathway. As an adaptor protein, importin α binds to target protein encompassing with NLS and then heterodimerizes with importin β, to assemble the heterotrimeric importin α/β/NLS-cargo complex that penetrates through NPC and delivers NLS-cargo into the nucleus (Christie et al., 2016; Nakada et al., 2017). Another crucial component of the nuclear import pathway, Ras-related nuclear protein (Ran), is a eukaryotic evolutionarily preserved small GTPase. Ran’s gradient regulates both effective export and import in GTP- and GDP-combined states between the cytoplasm and the nucleus. After attaching with RanGTP, the nuclear transport receptors binding cargo can be trafficked from the cytoplasm to the nucleus, while export receptors can release goods *via* binding to RanGTP and release them from the nucleus into the cytoplasm after GTP hydrolysis (Christie et al., 2016).

Nuclear export of proteins is largely fulfilled by a leucine-rich NES, bound by the major nuclear export receptor of the karyopherin-β family, chromosomal region maintenance 1 (CRM1) (exportin 1, XPO1). Transporter associated with antigen processing (TAP/NXF1) is also a major export receptor; both of them export mRNA from the nucleus to cytoplasm in metazoan cells and have been profoundly investigated. Although TAP is not a strong RNA-binding protein, it mainly binds to the Aly/REF mRNA adaptor protein, an element of the messenger ribonucleoprotein particles (mRNPs), which is carried out of the nucleus *via* direct combination with nucleoporins embedding into the nuclear pore (Moore and Rosbash, 2001). TAP/NXF1 can also improve the nuclear export of some proteins (Juillard et al., 2009; Ote et al., 2009; Li et al., 2011), whereas CRM1 can export hundreds of cargo proteins out of the nucleus by combining to their classical leucine-rich NESs (Fu et al., 2018). The CRM1-relied export pathway is widely utilized to export proteins and non-coding RNAs, including ribosomal RNAs (rRNAs) and small nuclear RNAs (snRNAs), while only a minority of cellular mRNAs employ this pathway (Fukuda et al., 1997; Fu et al., 2018). In addition to CRM1 and TAP, other exportins are also involved in the nuclear export process. Exportin 4 is in charge of the nuclear export of eukaryotic mothers against decapentaplegic homolog 3 (Smad3) and translation initiation factor 5A (eIF5A) (Kurisaki et al., 2006). Exportin 5 can mediate the export of dsRNA and precursor microRNA, while exportin-t can help export tRNA (Arts et al., 1998; Kim, 2004; Lund et al., 2004; Zeng and Cullen, 2004). Importantly, the exportin, cellular apoptosis susceptibility protein (CAS), can help export importin α for a new cycle of protein nuclear translocation (Kutay et al., 1997). Besides, exportin 7 is a nuclear export mediator with broad substrate specificity (Mingot et al., 2004).

It is well documented that the nuclear accumulation of herpes viral proteins is significant for virus propagation, assembly, and dissemination, whereas the nuclear transport mechanisms of the majority of virus-encoded proteins are less well explored. As a transcriptional co-activator of EBV nuclear antigen 2 (EBNA2), EBNA-LP interplays with importin α1 through functional NLS to facilitate its efficient nuclear localization for its interaction with EBNA2 (Nakada and Matsuura, 2017). The N-terminus functional NES of EBV early protein EB2 (also designated BMLF1, SM, or Mta) can advance the nucleocytoplasmic export of several early and late viral mRNAs (come from intron less genes) by precisely binding to TAP/NXF1, which is indispensable for the proliferation of infectious virions (Buisson et al., 1999; Juillard et al., 2009). EBNA1 can interact with importin α1 and importin α5 *via* its NLS to accelerate its nuclear import (Nakada et al., 2017), which may be fundamental for the sustainment, proliferation, and transcription of the EBV-positive tumor cells. BFLF2 is exhibited to be correlated with TAP for nuclear export and interplayed with importin α7, importin β1, and transportin-1 for its nuclear accumulation, which may be meaningful for the efficient viral DNA packaging and immediate release across the nuclear membrane (Li et al., 2018). Furthermore, BGLF4 protein kinase can promote the nuclear accumulation of a few non-NLS-containing EBV proteins, including major capsid protein (VCA) and the viral DNA replication enzymes BBLF2/3, BBLF4, and BSLF1 (Chang et al., 2015). However, the functional correlation of nucleocytoplasmic shuttling of most of the EBV proteins requires to be probed.

BLLF2 is an EBV-encoded protein with an unknown function. Our previous study manifested that BLLF2 localizes in the nucleus (Cai et al., 2017b); nonetheless, its subcellular localization’s definite mechanism was not well known. Our preliminary experiment established that BLLF2 could shuttle between the cytoplasm and nucleus, which propels us to investigate the nucleocytoplasmic transport mechanism of BLLF2.

## Materials and Methods

### Enzymes and Antibodies

All cloning enzymes were supplied by Thermo Scientific, with the exclusion of KOD-Plus-Neo DNA polymerase and T4 DNA Ligase, which were afforded by TOYOBO (Japan) and Takara (Beijing, China), respectively. Mouse anti-Flag monoclonal antibody (mAb) and rabbit anti-YFP polyclonal antibody (pAb) were offered by Abmart (Shanghai, China) and RayBiotech, respectively. Nonspecific IgG was purchased from Proteintech (Wuhan, China). Alkaline phosphatase (AP)-linked anti-rabbit IgG and anti-mouse IgG were provided by Cell Signaling Technology (MA, United States), and protein A/G PLUS-Agarose was bought from Santa Cruz (TX, United States).

### Construction of Expression Plasmids

Plasmid expressing EBV BLLF2 inserted into the C-terminus of EYFP (pEYFP-BLLF2) was constructed in our lab previously (Cai et al., 2017b). Diverse fragments and mutants of BLLF2 inserted into pEYFP-C1 (Clontech, BD Biosciences) were constructed with a similar method, as described previously (Li et al., 2015b, 2018). Plasmid expressing BLLF2-Myc (pBLLF2-Myc) and BLLF2-EYFP (pBLLF2-EYFP) were also generated by inserting a full-length BLLF2 fragment into the *Eco*RI- and *Bam*HI-digested vectors pMyc-N1 (regenerated from pEYFP-N1) and pEYFP-N1 [provided by Dr. Chunfu Zheng, School of Basic Medical Sciences, Fujian Medical University (Zhang et al., 2016; Huang et al., 2018; Yuan et al., 2018; You et al., 2019)], respectively. Furthermore, the short hairpin RNA (shRNA) for importin β1 (5’-CCA GTG TAG TTG TTC GAG ATA-3’) was inserted into pSUPER.retro.puro (shVector) (BD Biosciences) to construct pSUPER-shImportin β1 (shImportin-β1). The pSUPER-shRandom (shRandom) was described in our previous study (Chen et al., 2019; Wang et al., 2020). All constructed clones were validated by sequencing.

Besides, pseudorabies virus (PRV) UL31-EYFP, pEYFP-BFLF2, pUL4-EYFP, pTAP-mCherry, pCRM1-mCherry, pDN kα1-mCherry, pDN kβ1-mCherry, pRan-Q69L-mCherry, and pFlag-importin β2 expression plasmids were also constructed in our lab previously (Xing et al., 2011; Li et al., 2015b, 2018; Cai et al., 2017b). Plasmid pNucleolin-EGFP, a nucleolar marker, was provided by Dr. Johannes A. Schmid (Department of Vascular Biology and Thrombosis Research, University of Vienna Medical School and Competence Center Bio-Molecular Therapeutics). Expression plasmids of Flag-kα2 (importin α1), Flag-kα4 (importin α3), Flag-kα1 (importin α5), Flag-kα6 (importin α7), and pCMV9-3 × Flag-importin β1 were provided by Drs. Reinhard Depping (Department of Physiology, University of Lübeck), Yoshihiro Yoneda (Department of Biochemistry, Graduate School of Medicine, Osaka University), and Ben Margolis (Howard Hughes Medical Institute, University of Michigan Medical School), respectively. M9M-RFP and Bimax2-RFP were supplied by Dr. Nobuyuki Nukina (Laboratory for Structural Neuropathology, Brain Science Institute).

### Cell Culture, Transfection, and Subcellular Localization

HEK393T cells, COS-7 cells, and NIH3T3 cells were cultured and transfected by employing polyethylenimine (Sigma, Shanghai, China), as previously described (Chen et al., 2019). 24 h post-transfection, cells were washed with PBS and then dyed with 4′,6′-diamidino-2-phenylindole (DAPI) (Thermo Fisher Scientific, United States) for 5 min to view the nucleus (blue). Finally, the stained cells were inspected by a fluorescence microscope.

### Confocal Microscopy Analysis

COS-7 cells, cultured on cover-slips in the 24-well plate (Corning, United States), were co-transfected with the indicated expression plasmids for 24 h. Transfected cells were then fixed with 4% paraformaldehyde (Tianjun biotechnology, China) for 30 min. Subsequently, the fixed cells were washed three times with PBS (Boster, Wuhan, China), followed by staining with DAPI for 5 min at 37°C. Then, the stained cells were placed on the microscope slides (Biosharp, Shanghai, China) for confocal microscope analysis. Samples were assayed through a Leica confocal laser scanning microscope (Leica SP8) using 63 × 1.4 NA immersion oil lenses, with excitation wavelength at 512 nm for YFP/EGFP, 405 nm for DAPI, and 555–580 nm for mCherry/RFP.

### Interspecies Heterokaryon Assays

The interspecies heterokaryon assays were performed following previous studies (Tao and Levine, 1999; Rodriguez and Henderson, 2000; Xu et al., 2002; Tanno et al., 2007; Juillard et al., 2009; Li et al., 2011; Zheng et al., 2019). In short, monkey COS-7 cells were seeded in six-well plates and transfected with the indicated plasmids. 18 h post-transfection, mouse NIH3T3 cells were added into the COS-7 cells containing cycloheximide (50 μg/ml, inhibiting the synthesis of new proteins) (Sigma, Shanghai, China), which was used to prevent the residual target plasmid from entering NIH3T3 cells for expression, to help us analyze whether the target protein can shuttle from COS-7 cells to NIH3T3 cells. In the experiments, cells were treated with or without 20 ng/ml leptomycin B (LMB) (Sigma, Shanghai, China). 4 h later, polyethylene glycol (Sigma, Shanghai, China) was employed to fuse COS-7 cells with NIH3T3 cells. After hatching for 1 h, cells were stained with DAPI and imaged by fluorescence microscopy.

### Co-immunoprecipitation and Western Blot Assays

Co-immunoprecipitation (Co-IP) and Western blot (WB) assays were performed as described previously (Cai et al., 2013, 2016a; Su and Zheng, 2017; Xu et al., 2017; Ye et al., 2017). Briefly, HEK293T cells co-transfected with different expression plasmid combinations were collected and lysed with 600 to 800 μl of lysis buffer on ice for 30 min and then centrifuged at 12,000 *g* at 4°C for 15 min. For each Co-IP, supernatants were immunoprecipitated with anti-YFP pAb, anti-Flag mAb, or nonspecific IgG at 4°C for 2 to 4 h, and then incubated overnight with protein A/G beads. The beads were then washed at least four times with PBS buffer at 2500 *g* at 4°C for 5 min. Subsequently, the complex with SDS-PAGE loading buffer was boiled for 10 min and subjected to SDS-PAGE/WB analysis after centrifuging at 12,000 *g* for 5 min. For WB analysis, the immunoprecipitated proteins were stained with the indicated Abs. Cell lysates were also directly subjected to WB analysis to verify the expression of specific proteins.

## Results

### Nucleocytoplasmic Shuttling of BLLF2

**FIGURE 1:**
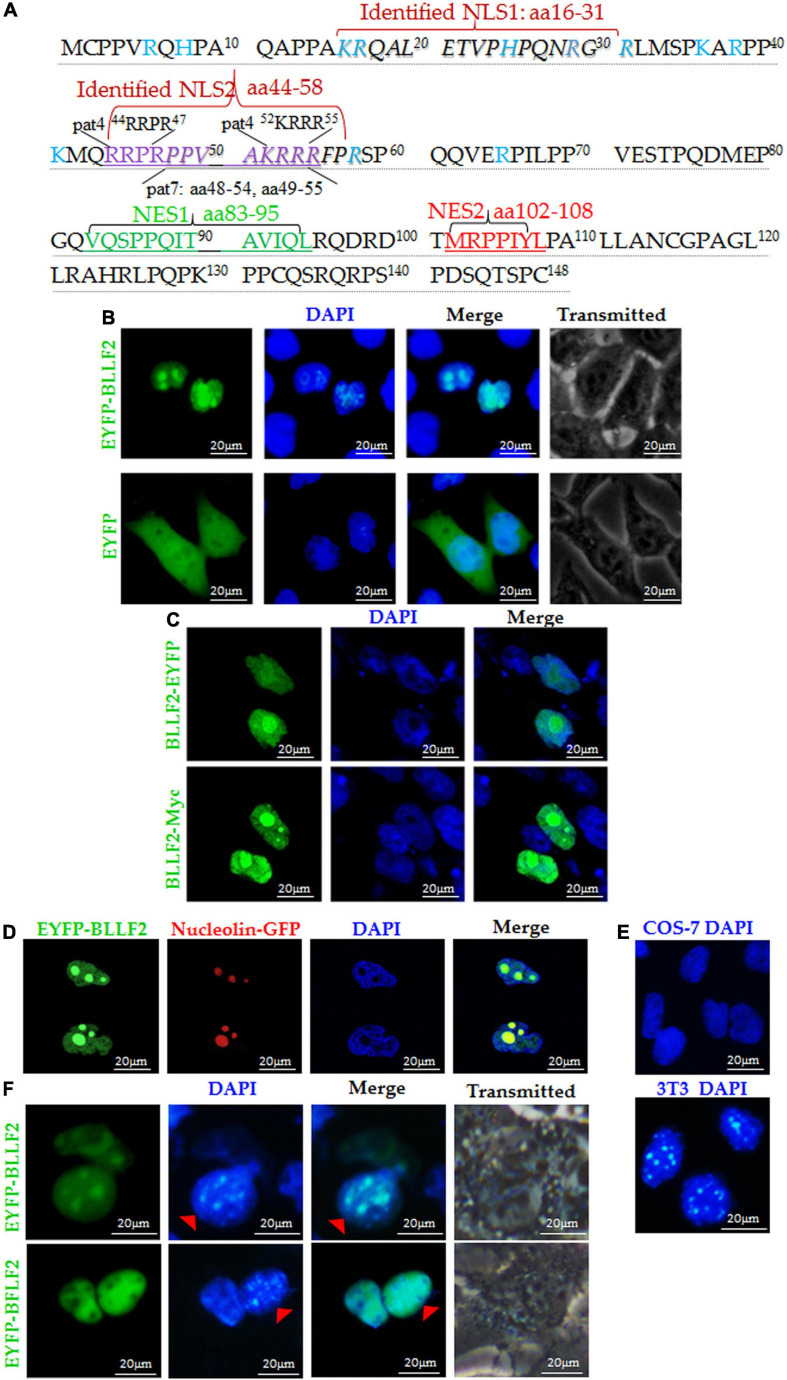
Subcellular localization and nucleocytoplasmic shuttling of BLLF2. **(A)** Potential NESs and NLSs of BLLF2 were predicted by the bioinformatics software NetNES 1.1 and PSORT II, respectively. Proteins assigned for trafficking into the nucleus encompass aa targeting sequences termed NLSs (Lange et al., 2007), and proteins bound for delivery out of the nucleus consisting of aa targeting sequences named NESs (La Cour et al., 2004). The basic aas are arginine (R), histidine (H), and lysine (K). PSORT II adopts the following two standards to dissect target protein: four-residue pattern (termed “pat4”) formed by four basic aa (K or R), or formed by three basic aa (K or R) and either H or P; the other (termed “pat7”) is a pattern beginning with P and followed within three residues by a basic segment including three K/R residues out of four. The identified NLSs ^16^KRQALETVPHPQNRGR^31^ (NLS1) and ^44^RRPRPPVAKRRRFPR^58^ (NLS2) were also indicated. **(B)** Subcellular distributions of EYFP-BLLF2 and EYFP vector in COS-7 cells. **(C)** Subcellular distributions of BLLF2-EYFP and BLLF2-Myc in COS-7 cells. **(D)** Co-expression of EYFP-BLLF2 and pNucleolin-EGFP was observed in COS-7 cells. **(E)** COS-7 and NIH3T3 cells were stained with DAPI. NIH3T3 cells were identified by their speckled nuclei. **(F)** Nucleocytoplasmic shuttling of BLLF2 was demonstrated by interspecies heterokaryon assays. EYFP-BFLF2 was used as nucleocytoplasmic shuttling positive control. COS-7 and NIH3T3 cells were discriminated against by nuclear staining with DAPI. NIH3T3 cells were identified by their speckled nuclei (red arrowhead). All scale bars indicate 20 μm.

Our previous study revealed that BLLF2 is located in the nucleus (Cai et al., 2017b). To dissect the subcellular transport mechanism of BLLF2, bioinformatics analysis was initially implemented and showed that BLLF2 possesses four potential NLS motifs (pat4 ^44^RRPR^47^ and ^52^KRRR^55^, and pat7 ^48^PPVAKRR^54^ and ^49^PVAKRRR^55^) and two supposed NES, namely, NES1 (^83^VQSPPQITAVIQL^95^) and NES2 (^102^MRPPIYL^108^) (Figure 1A). Subsequently, BLLF2 was again confirmed completely located in the nucleus in COS-7 cells transfected with EYFP-BLLF2 plasmid (Figure 1B). To exclude the influence of the big tag EYFP on the subcellular localization of BLLF2, BLLF2-EYFP and BLLF2-Myc expression plasmids were also constructed, and fluorescence microscope demonstrated that the subcellular localization patterns of BLLF2-EYFP and BLLF2-Myc were similar to that of EYFP-BLLF2 (Figure 1C). Besides, co-expression of nucleolar marker pNucleolin-EGFP with pEYFP-BLLF2 showed that BLLF2 is also located in the nucleolus (Figure 1D).

A nuclear localization protein equipped with functional NES and NLS can be theoretically transported from a donor to a recipient nucleus (Agutter and Prochnow, 1994). Concerning the heterokaryon assays, the shuttling characteristic of a certain protein can be established through the species-specific DAPI staining fashions of the nucleus under a fluorescence microscope, with speckles in the nucleus of mouse NIH3T3 cells (Figure 1E; Li et al., 2011). Since BLLF2 holds predicted NES and NLS, we wondered if BLLF2 can shuttle between the cytoplasm and nucleus. As a result (Figure 1F), COS-7 cells expressing EYFP-BLLF2 were fused with a considerable number of NIH3T3 cells in the presence of the protein synthesis inhibitor cycloheximide. After fusing, BLLF2 could also be detected with a typical speckled fashion in the nucleus of NIH3T3 cells. As the nucleocytoplasmic shuttling positive control (Li et al., 2018), EYFP-BFLF2 could shuttle between COS-7 cells and NIH3T3 cells, suggesting that BLLF2 is a genuine nucleocytoplasmic shuttling protein.

### Identification of the Functional NES in BLLF2

**FIGURE 2:**
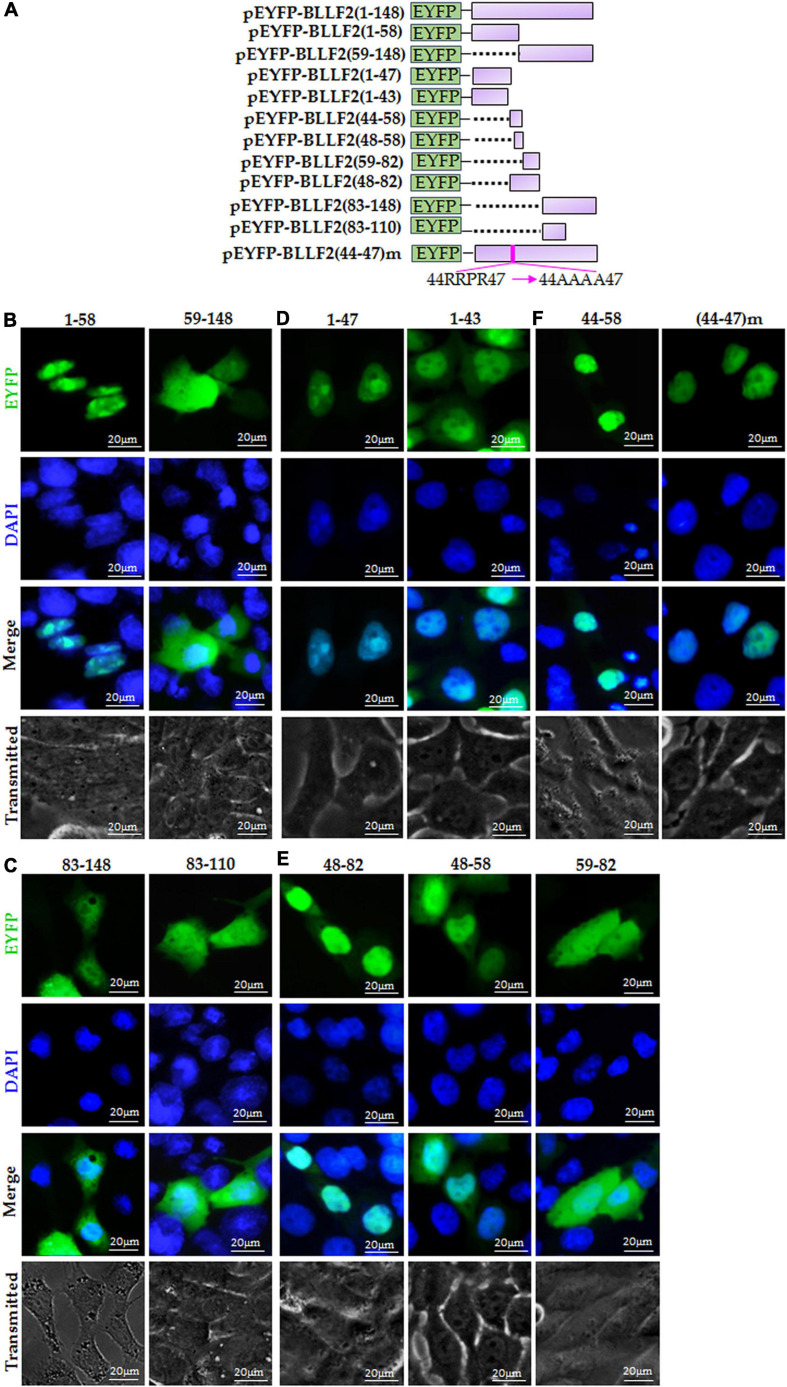
Identification of the predicted NES and functional NLS in BLLF2. **(A)** Schematic diagram of constructs encoding EYFP-tagged wild-type BLLF2 and its deletion mutants aa1–43, 1–47, 1–58, 44–58, 48–58, 48–82, 59–82, 59–148, 83–148, 83–110, and full-length mutant BLLF2(44–47)m. **(B)** Intracellular localization of deletion mutants BLLF2(1–58) and BLLF2(59–148) in COS-7 cells. **(C)** Intracellular localization of deletion mutants BLLF2(83–143) and BLLF2(83–110) in COS-7 cells. **(D)** Intracellular localization of deletion mutants BLLF2(1–47) and BLLF2(1–43) in COS-7 cells. **(E)** Intracellular localization of deletion mutants BLLF2(48–82), BLLF2(48–58), and BLLF2(59–82) in COS-7 cells. **(F)** Intracellular localization of deletion mutant BLLF2(44–58) and full-length mutant BLLF2(44–47)m in COS-7 cells. All scale bars indicate 20 μm.

Based on the predicted motifs of NESs and NLSs, full-length BLLF2 was firstly cut into two fragments [amino acids (aa)1–58 and aa59–148] and then fused to the C-terminus of EYFP (Figure 2A) to detect their subcellular localizations. As shown in Figure 2B, aa1–58 was perfectly located in the nucleus and nucleolus, while aa59–148 displayed disperse dissemination throughout the cytoplasm and nucleus. These results disclosed that aa1–58 contains functional NLS, and aa59–148 may not contain functional NES.

To further find out the functional NES of BLLF2, pEYFP-BLLF2(83–148) was constructed (Figure 2A) and transfected into COS-7 cells, which had a similar subcellular localization pattern with that of aa59–148 (Figure 2B). Next, aa83–148 was shortened to aa83-110 (contains predicted NES1 and NES2) to create pEYFP-BLLF2(83–110) (Figure 2A), and the result uncovered that aa83–110 also homogeneously localized throughout the cytoplasm and nucleus (Figure 2C). These results confirmed that the predicted NES1 and NES2 are nonfunctional.

### Identification of the Functional NLS in BLLF2

To analyze whether the predicted pat4 (^44^RRPR^47^) is a functional NLS, pEYFP-BLLF2(1–47) and pEYFP-BLLF2(1–43) were constructed (Figure 2A) and tested in COS-7 cells. As a result, aa1–47 was entirely located in the nucleus and nucleolus (Figure 2D), indicating that ^44^RRPR^47^ may take effect on the nucleolus localization, or aa1–43 contains functional NLS. As expected, aa1–43 was also located in the nucleus without nucleolus (Figure 2D), proving that aa1–43 contains functional NLS, which may be composed by ^25^HPQNRGRLMSPKARPPK^41^.

For the sake of ascertaining whether the predicted pat4 (^52^KRRR^55^) and pat7 (^48^PPVAKRR^54^ and ^49^PVAKRRR^55^) in aa48–58 of BLLF2 are functional, pEYFP-BLLF2(48–82), pEYFP-BLLF2(48–58), and pEYFP-BLLF2(59–82) were constructed (Figure 2A) and assessed in COS-7 cells. As shown in Figure 2E, aa48–82 was predominantly located in the nucleus without nucleolus, demonstrating that ^48^PPVAKRRR^55^ may have a nuclear localization effect for aa48–82. Moreover, aa48–58 was mostly located in the nucleus, indicating that aa48–58 can function as an NLS. However, aa59–82 showed a pan-cellular localization pattern, suggesting that this region does not contain functional NLS.

To validate whether pat4 (^44^RRPR^47^) of aa44–58 has a role in nucleolus localization, pEYFP-BLLF2(44–58) was constructed (Figure 2A) and examined in COS-7 cells. The result showed that aa44–58 was located in the nucleus and nucleolus (Figure 2F), suggesting that pat4 (^44^RRPR^47^) may be a functional nucleolus localization signal and ^44^RRPRPPVAKRRRFPR^58^ is an authentic NLS. To certify the nucleolus localization role of ^44^RRPR^47^, ^44^RRPR^47^ was mutated to ^44^AAAA^47^ in the full length of BLLF2 and then fused to the C-terminus of EYFP to yield pEYFP-BLLF2(44–47)m. As a result, the nuclear and nucleolus localization pattern of BLLF2 was alternated into pan-nuclear localization without nucleolus (Figure 2F). These data confirmed that 44RRPR47 has a nucleolus localization function.

**FIGURE 3:**
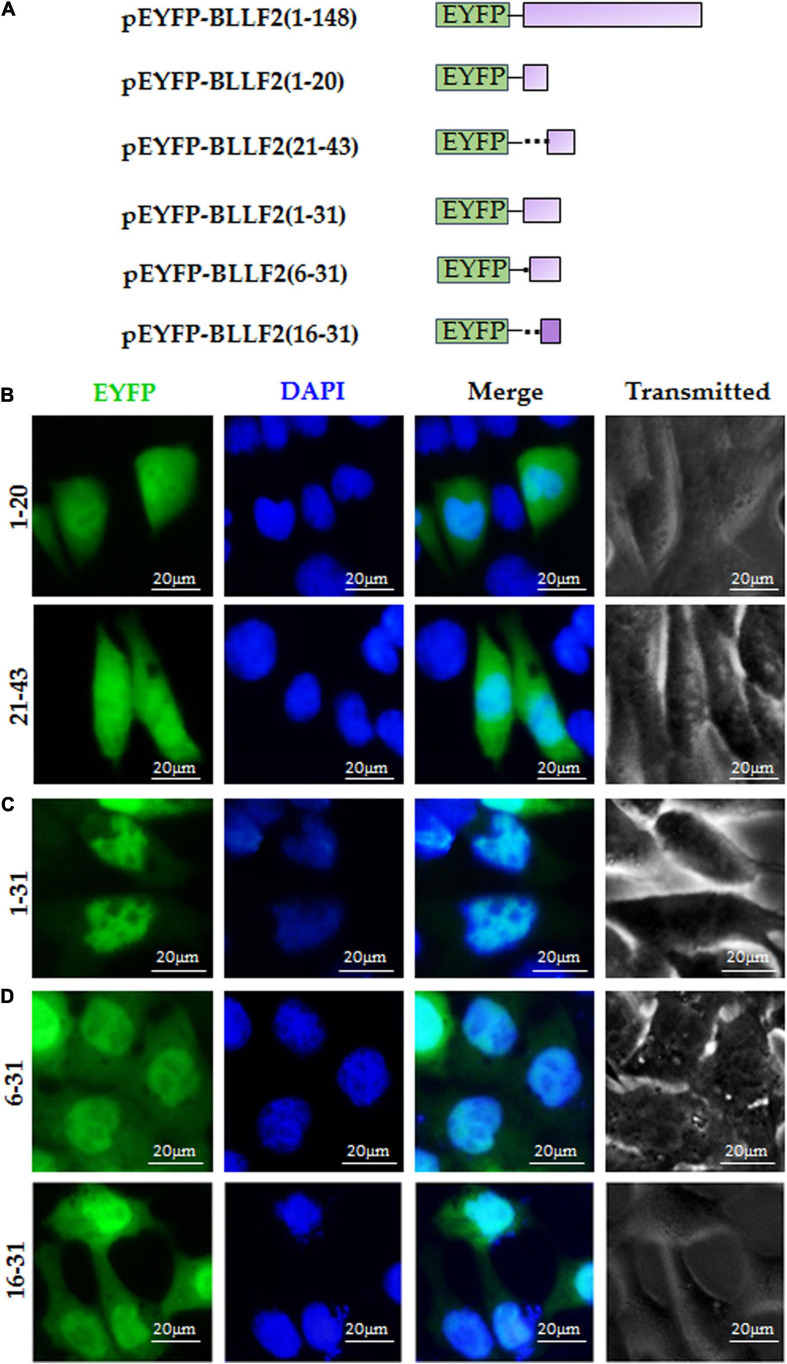
aa16–31 is another functional NLS of BLLF2. **(A)** Schematic diagram of constructs encoding EYFP-tagged wild-type BLLF2 and its deletion mutants aa1–20, 21–43, 1–31, 6–31, and 16–31. **(B)** Intracellular localization of deletion mutants BLLF2(1–20) and BLLF2(21–43) in COS-7 cells. **(C)** Intracellular localization of deletion mutant BLLF2(1–31) in COS-7 cells. **(D)** Intracellular localization of deletion mutants BLLF2(6–31) and BLLF2(16–31) in COS-7 cells. All scale bars indicate 20 μm.

Next, to further inspect the functional NLS of aa1–43, pEYFP-BLLF2(1–20) and pEYFP-BLLF2(21–43) were constructed (Figure 3A) and assayed in COS-7 cells. As shown in Figure 3B, both fluorescences of aa1–20 and aa21–43 had similar subcellular localization patterns with that of the EYFP control, indicating that these two regions do not contain functional NLS. Furthermore, when aa1–20 was extended to aa1–31, aa1–31 showed conspicuous nuclear localization (Figure 3C), suggesting that aa1–31 possesses functional NLS, which may be located in aa6–31 or aa16–31.

To eventually determine the minimum NLS region of aa1–43, pEYFP-BLLF2(6–31) and pEYFP-BLLF2(16–31) were constructed (Figure 3A) and transfected into COS-7 cells. As a result, aa6–31 and aa16–31 showed parallel subcellular localization to that of aa1–31, with dominant nuclear localization (Figure 3D), disclosing aa16–31 is another functional NLS of BLLF2.

### Nuclear Import Mechanism of BLLF2

**FIGURE 4:**
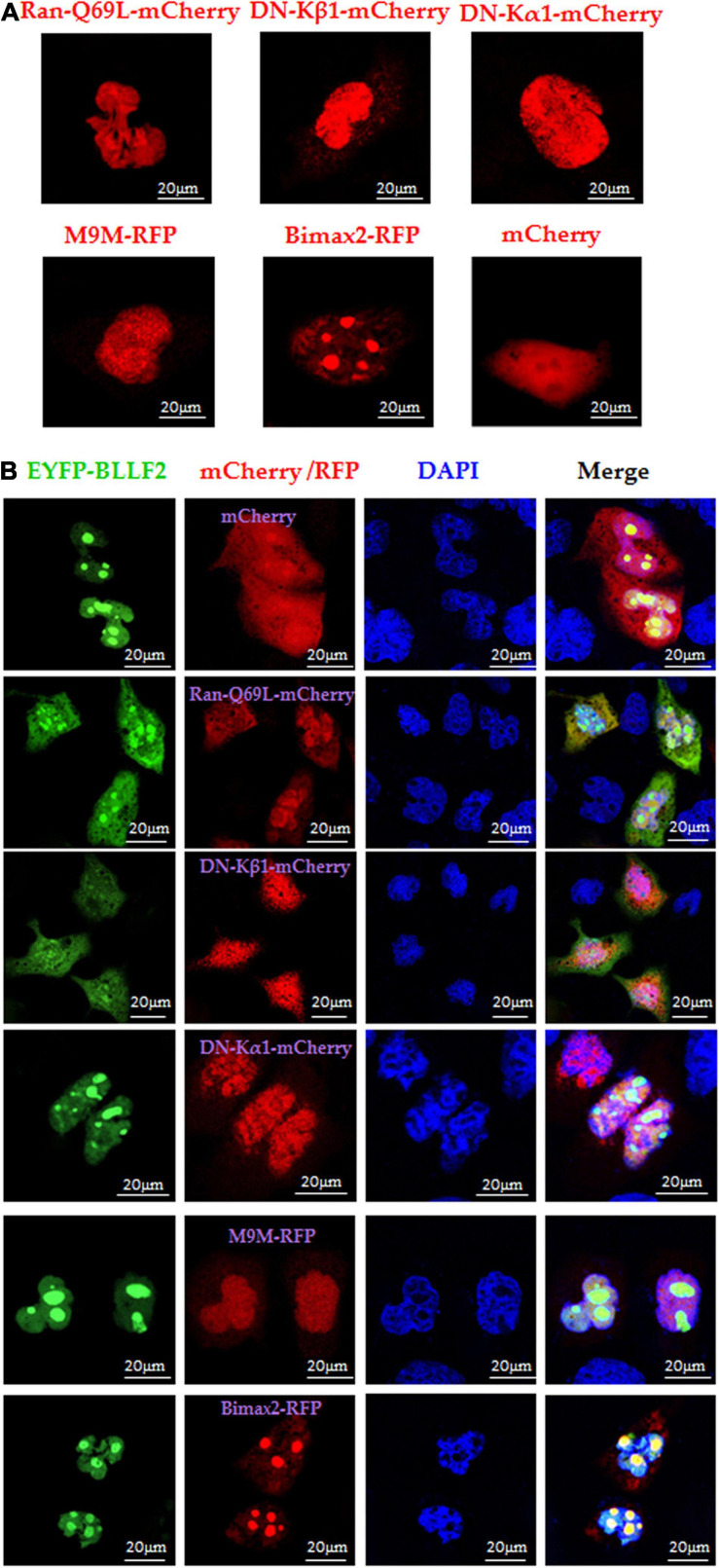
Nuclear import mechanism of BLLF2. **(A)** Individual subcellular localization of Ran-Q69L-mCherry, DN kα1-mCherry, DN kβ1-mCherry, M9M-RFP, Bimax2-RFP, or mCherry vector in COS-7 cells. **(B)** Co-expression of Ran-Q69L-mCherry/EYFP-BLLF2, DN kα1-mCherry/EYFP-BLLF2, DN kβ1-mCherry/EYFP-BLLF2, M9M-RFP/EYFP-BLLF2, Bimax2-RFP/EYFP-BLLF2, or mCherry/EYFP-BLLF2 in COS-7 cells. All scale bars indicate 20 μm.

Ran (Ras-associated nuclear protein), a small GTPase belonging to the RAS superfamily, is specialized and crucial for the nuclear accumulation of proteins with a canonical NLS (Coppola et al., 2018). Here, dominant-negative (DN) RanGTP containing Q69 mutation (Ran-Q69L), without the competence of GTP hydrolysis (Li et al., 2011), was applied to dissect whether Ran is vital for the nuclear trafficking of BLLF2. Compared to the cells co-transfected with mCherry vector and pEYFP-BLLF2, the nuclear import of BLLF2 was undoubtedly confined in cells co-expressing Ran-Q69L-mCherry and EYFP-BLLF2 (Figure 4B), suggesting that the nuclear translocation of BLLF2 is Ran-relied and requires Ran GTP hydrolysis.

It is universally established that the importin α/β heterodimer can discern canonical NLS and facilitate the nucleocytoplasmic transport of particular target proteins (Lott and Cingolani, 2011). To resolve which receptor takes part in the nuclear translocation of BLLF2, DN importin α5 [kα1, flawed in the binding to importin β (Chi et al., 1997)] and DN importin β1 [kβ1, faulty in associating with Ran (Chi et al., 1997)] were exploited. Moreover, the nuclear import inhibitors of M9M (specifically impedes importin β2 to attach to NLS) and Bimax2 [suppresses the functions of importin α1, α3, and α7 (Li et al., 2011)] were also applied. Compared to the negative controls of DNs or competitive inhibitors (Figure 4A), BLLF2 was relocalized to the cytoplasm by DN kβ1, but not DN kα1, M9M, Bimax2, or mCherry (Figure 4B), suggesting that BLLF2 may be transported into the nucleus *via* Ran- and importin β1-dependent pathway.

### Nuclear Export Mechanism of BLLF2

**FIGURE 5:**
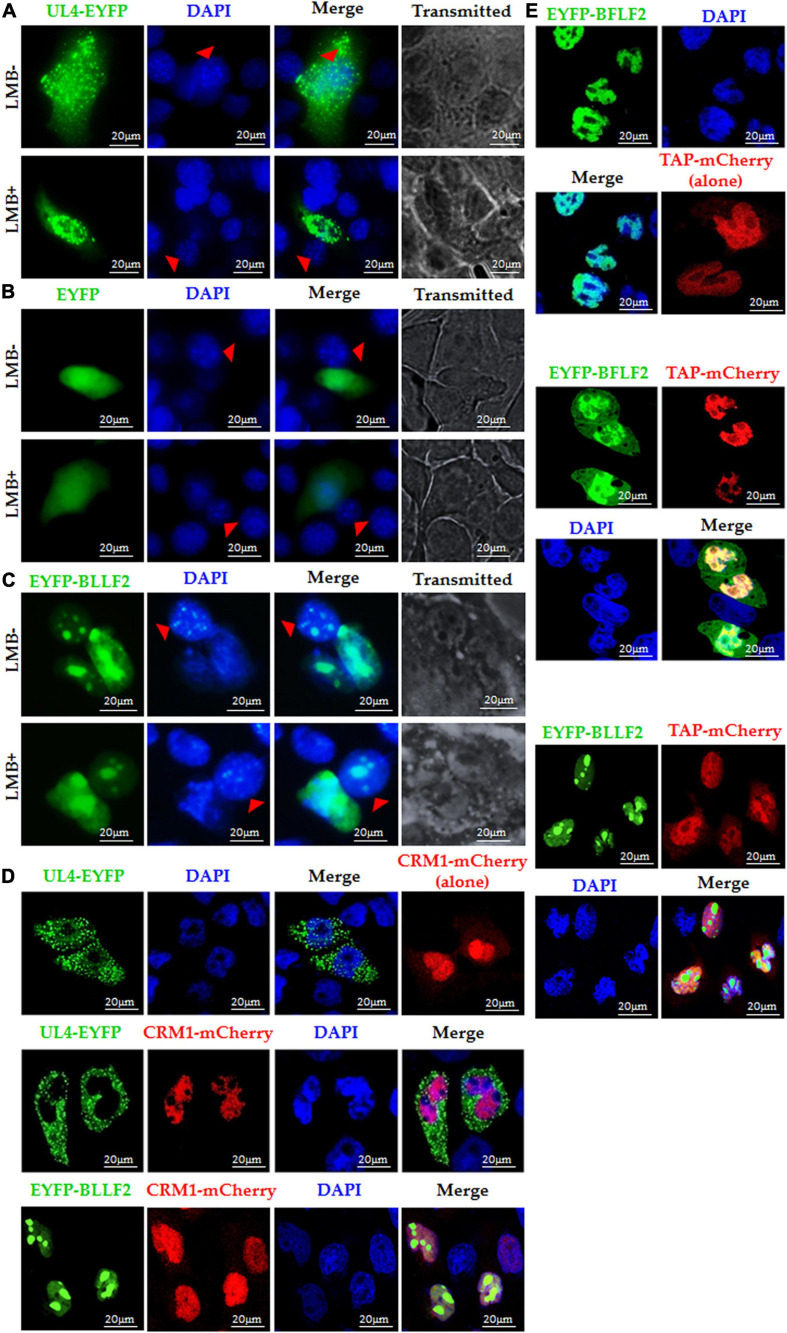
Nuclear export mechanism of BLLF2. Interspecies heterokaryon assays were performed to analyze the nuclear export of BLLF2. Mouse NIH3T3 cells were plated onto the CRM1-dependent positive control UL4 **(A)**, CRM1-independent negative control EYFP vector **(B)**, or pEYFP-BLLF2 **(C)** transfected COS-7 cells, with or without LMB treatment, as described in Section “Materials and Methods.” Cells were then stained with DAPI and imaged by fluorescence microscopy. NIH3T3 cells were identified by their speckled nuclei (red arrowhead). **(D)** COS-7 cells were individually transfected with UL4-EYFP or co-transfected with expression plasmids CRM1-mCherry/UL4-EYFP or CRM1-mCherry/EYFP-BLLF2 and then examined by confocal microscopy. **(E)** COS-7 cells were individually transfected with the TAP-dependent positive control EYFP-BFLF2 or co-transfected with expression plasmids TAP-mCherry/EYFP-BFLF2 or TAP-mCherry/EYFP-BLLF2, and then examined by confocal microscopy. All scale bars indicate 20 μm.

Chromosomal region maintenance 1 (Exportin1/XPO1), a member of the importin β family, mediates the nuclear export of proteins by combining them to their classical NESs (Fu et al., 2018). Therefore, we continued to assess whether the nuclear export of BLLF2 can be blocked by CRM1 specific inhibitor leptomycin B (LMB) (Li et al., 2011). As CRM1-dependent positive control (Pan et al., 2011), the nuclear accumulation of HSV-1 UL4 was inhibited by LMB treatment (Figure 5A), whereas the CRM1-independent negative control of the EYFP vector was incapable of achieving the nucleocytoplasmic shuttling with or without the presence of LMB (Figure 5B). Upon LMB treatment, EYFP-BLLF2 was also transported from monkey nuclei to mouse nuclei (Figure 5C), indicating that BLLF2 can shuttle between varied cells, and the nuclear export of BLLF2 may take place independently on CRM1.

It is reported that CRM1 overexpression can advance the nuclear export of CRM1-dependent proteins (Li et al., 2018). As a result, the nuclear export of the CRM1-dependent positive control UL4 (Pan et al., 2011) was promoted by co-expression of CRM1-mCherry, yet BLLF2 remain thoroughly located in the nucleus and nucleolus in the existence of CRM1 when COS-7 cells were co-transfected with pCRM1-mCherry and pEYFP-BLLF2 (Figure 5D), certifying CRM1 is not fundamental for the nuclear export of BLLF2.

Besides CRM1, TAP (NXF1), the critical mRNA export receptor, is also related to the nuclear export of distinct proteins (Mamon et al., 2017), which can also expedite the nuclear export of TAP-relied protein when it is overexpressed with the target protein (Li et al., 2018). As shown in Figure 5E, the nuclear export of the TAP-dependent positive control BFLF2 (Li et al., 2018) was boosted by co-expression of TAP-mCherry, whereas TAP could not transport BLLF2 from the nucleus to cytoplasm when COS-7 cells were co-transfected with EYFP-BLLF2 and TAP-mCherry expression plasmids, testifying that the nuclear export of BLLF2 was also independent on TAP.

### BLLF2 Binds to Importin β1

**FIGURE 6:**
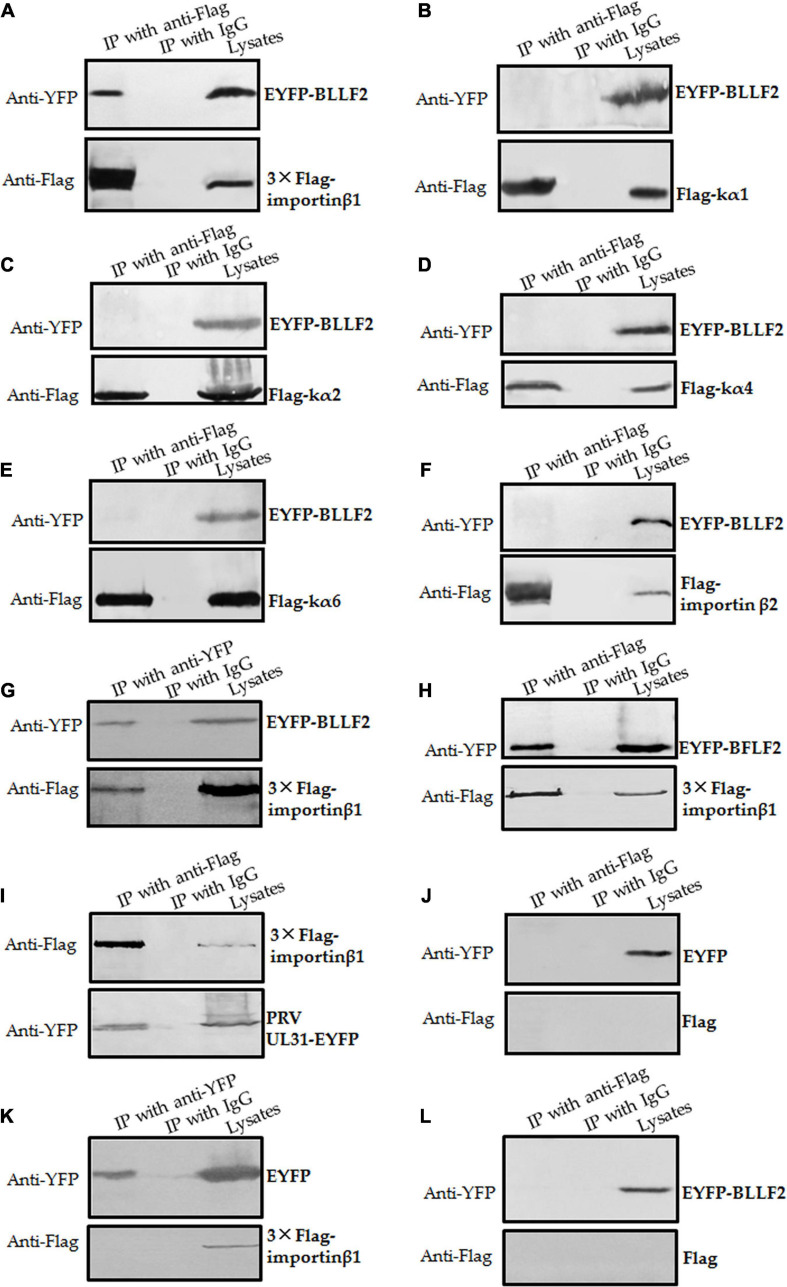
BLLF2 binds to importin β1. **(A–G,L)** Co-IP analysis of BLLF2 with importin β1 **(A,G)**, importin α5 (kα1) **(B)**, importin α1 (kα2) **(C)**, importin α3 (kα4) **(D)**, importin α7 (kα6) **(E)**, importin β2 **(F)**, or Flag vector **(L)**. HEK293T cells were co-transected with expression plasmids combination of 3 × Flag-importin β1/EYFP-BLLF2 **(A,G)**, Flag-kα1/EYFP-BLLF2 **(B)**, Flag-kα2/EYFP-BLLF2 **(C)**, Flag-kα4/EYFP-BLLF2 **(D)**, Flag-kα6/EYFP-BLLF2 **(E)**, Flag-importin β2/EYFP-BLLF2 **(F)**, or Flag vector/EYFP-BLLF2 **(L)** for 24 h; cells were subsequently lysed and Co-IPed with anti-Flag mAb **(A–F,L)** or anti-YFP pAb **(G)** or control IgG, and then WB analysis was carried out with the indicated Abs. **(H,I)** Co-IP analysis of importin β1 with BFLF2 **(H)** or PRV UL31 **(I)**. HEK293T cells were co-transected with expression plasmids combination of 3 × Flag-importin β1/EYFP-BFLF2 **(H)** or 3 × Flag-importin β1/PRV UL31-EYFP **(I)** for 24 h, and cells were then lysed and Co-IPed with anti-Flag mAb or control IgG, and then WB analysis was carried out with the indicated Abs. **(J,K)** Co-IP analysis of EYFP vector with Flag vector **(J)** or importin β1 **(K)**. HEK293T cells were co-transected with expression plasmids combination of Flag vector/EYFP vector **(J)** or 3 × Flag-importin β1/EYFP vector **(K)** for 24 h; cells were then lysed and Co-IPed with anti-Flag mAb **(J)** or anti-YFP **(K)** or control IgG, and then WB analysis was carried out with the indicated Abs.

To further test the assumption mentioned above, the interactions of BLLF2 with human importin α/β molecules, importin α1 (kα2), importin α3 (kα4), importin α5 (kα1), importin α7 (kα6), importin β1, and importin β2, were inspected. Plasmid expressing EYFP-BLLF2, EYFP-BFLF2, PRV UL31-EYFP, or EYFP vector was co-transfected with Flag-tagged importins or Flag vector into HEK293T cells for 24 h and then cell lysates were harvested for Co-IP assays. In comparison to the negative IgG (Figure 6), EYFP-BLLF2 was perfectly Co-IPed with 3 × Flag-importin β1 (by using anti-Flag mAb) (Figure 6A), rather than kα1 (Figure 6B), kα2 (Figure 6C), kα4 (Figure 6D), kα6 (Figure 6E), or importin β2 (Figure 6F). To verify the interaction between EYFP-BLLF2 and 3 × Flag-importin β1, reversed Co-IP was performed with anti-YFP antibody, and the result showed that 3 × Flag importin β1 could be effectively pulled down by EYFP-BLLF2 (Figure 6G). As positive controls (Li et al., 2015a, 2018), EYFP-BFLF2 and PRV UL31-EYFP could be Co-IPed with anti-Flag mAb when HEK293T cells were co-transfected with plasmids expressing EYFP-BFLF2/3 × Flag-importin β1 (Figure 6H) or PRV UL31-EYFP/3 × Flag-importin β1 (Figure 6I). However, no EYFP protein (Figure 6J), 3 × Flag-importin β1 (Figure 6K), or EYFP-BLLF2 (Figure 6L) was Co-IPed with anti-Flag mAb (Figure 6J), anti-YFP pAb (Figure 6K), or anti-Flag mAb (Figure 6L) when HEK293T cells were co-transfected with EYFP vector and Flag vector (Figure 6J), EYFP vector and 3 × Flag-importin β1 (Figure 6K), or EYFP-BLLF2 and Flag vector (Figure 6L), indicating that BLLF2 can interplay with the nuclear import receptor importin β1, but not importin α1, α3, α5, α7, or importin β2.

### Establishment of the Nuclear Translocation Mechanism of BLLF2

**FIGURE 7:**
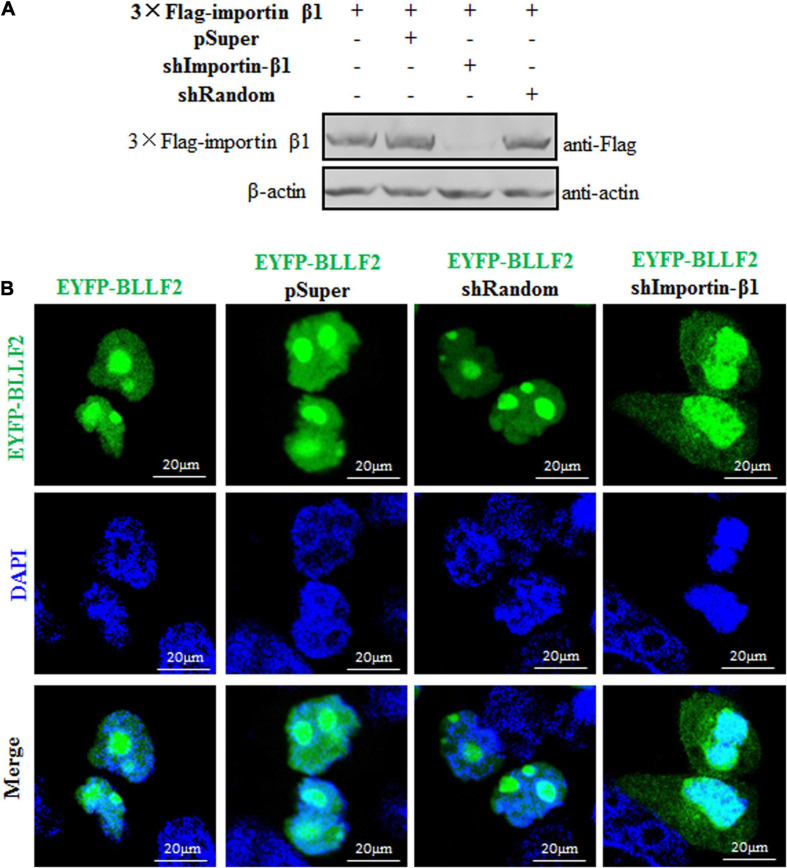
Subcellular localization of BLLF2 when importin β1 was knocked down. **(A)** Validation of knockdown efficiency of the constructed shImportin-β1 expression plasmid. HEK293T cells were transfected with 3 × Flag-importin β1 expression plasmid or co-transfected with the expression plasmids combination of 3 × Flag-importin β1/pSuper, 3 × Flag-importin β1/shRandom, or 3 × Flag-importin β1/shImportin-β1 for 24 h. Then, cells were lysed, and WB was carried out with anti-Flag mAb. β-actin was used as a loading control. **(B)** pEYFP-BLLF2 was transfected into COS-7 cells, or pEYFP-BLLF2 was co-transfected with pSuper, shRandom, or shImportin-β1 expression plasmid into COS-7 cells. At 24 h post-transfection, confocal fluorescence microscopy was executed to examine the subcellular localization of BLLF2.

To finally verify the nuclear translocation mechanism of BLLF2, an shRNA expression plasmid was created to knock down the expression of importin β1. Compared to the pSuper vector and shRNA control vector (shRandom), shImportin-β1 could efficiently downregulate the expression of importin β1 (Figure 7A), suggesting that the shRNA expression plasmid of importin β1 was successfully constructed. Then, expression plasmid of pSuper vector, shRandom, or shImportin-β1 was co-transfected with EYFP-BLLF2 into COS-7 cells to assay whether shImportin-β1 can affect the nuclear trafficking of BLLF2. As shown in Figure 7B, the nuclear accumulation of BLLF2 was not disturbed by pSuper or shRandom. However, this nuclear translocation was remarkably impeded when importin β1 was knocked down (Figure 7B), resulting in its prominent disruption of nucleolus localization and as a result of cytoplasm translocation, proving that BLLF2 could be transported into the nucleus *via* importin β1.

## Discussion

Subcellular distribution of nuclear protein was first shown to shuttle between the cytoplasm and the nucleus by transplantation experiment in *Amoeba proteus* (Goldstein, 1958), and an increasing number of proteins are proved to have the ability of nucleocytoplasmic shuttling (Borer et al., 1989). Nonetheless, the nucleocytoplasmic shuttling feature of protein from higher eukaryotic cells is shown based on a cell fusion experiment. In interspecies heterokaryon assays, we found that the EBV encoded protein BLLF2 with unknown function could locate in the nucleus and nucleolus and shuttle between the cytoplasm and nucleus.

When herpes virus invades cells, some virus encoded proteins are delivered into the cytoplasm, which may stay in the cytoplasm or transfer into the nucleus to achieve their corresponding functions, such as inhibiting the transcription and translation of the host cells, restraining the host innate immunity, etc., to facilitate the propagation of the virus. After replication, the virus will synthesize a series of structural proteins to assemble progeny virions, which also play different roles in the viral life cycle. In the case of transient transfection, the newly synthesized BLLF2 fusion protein will also locate in the specific cell compartments to perform its function(s), simulating the function(s) and/or characteristics of BLLF2 to a certain extent during EBV infection. As we have known, a specific antibody is a key tool to investigate the function of the target protein. In our previous experiment design, we had considered we could use a specific BLLF2 antibody to detect the time-course expression and subcellular localization of BLLF2 during EBV lytic infection-induced from EBV latent cells, which can be used to analyze the correlation between the function(s) and characteristics of BLLF2 during EBV lytic infection and BLLF2 transient expression. We had tried to induce the expression of BLLF2 in prokaryotes (*Escherichia coli*) to prepare its antibody by using the mature antibody preparation technology in our lab (Li et al., 2014; Cai et al., 2015), but the expression of BLLF2 could not be effectively induced in *E. coli*. Due to the codon preference of BLLF2, it may be different from that of *E. coli*, which makes it impossible for us to purify BLLF2 protein and prepare its antibody. Besides, there is no commercial BLLF2 antibody available at present. Thus, it cannot directly analyze the effect of nuclear translocation of BLLF2 on viral replication during EBV lytic infection.

It is well known that the herpesvirus encoded genes can be divided into IE gene, E gene, and L gene according to the order of gene expression time. During infection, IE genes are firstly expressed to regulate the expression of other genes predominantly, E genes encode some enzymes, DNA binding proteins, or other proteins to regulate viral DNA replication, and L genes generally encode proteins that constitute the components of progeny virions (Pereira et al., 1977; Hammerschmidt and Sugden, 2013). Because BLLF2 is an EBV encoded E gene, it can regulate the transcriptional expression of EBV L genes (Yuan et al., 2006); we, therefore, speculate that the nuclear accumulation of BLLF2 during EBV infection must play an important for the production of late proteins and progeny virions of EBV, but this hypothesis needs to be verified in the future.

For accomplishing nucleocytoplasmic shuttling, nuclear localization of a specific protein demands functional NES and NLS engagement. Bioinformatics analysis revealed that BLLF2 carries four potential NLS motifs (pat4 ^44^RRPR^47^ and ^52^KRRR^55^, and pat7 ^48^PPVAKRR^54^ and ^49^PVAKRRR^55^) and two potential NES ^83^VQSPPQITAVIQL^95^ (NES1) and ^102^MRPPIYL^108^ (NES2). In the present study, aa1–82 was divided into two regions aa1–47 and aa48–82. In aa1–47, aa16–31 was identified as a genuine functional NLS (NLS1). Moreover, aa48–58 was also established as a functional NLS in aa48–82, and aa44–47 exerted a substantial role in the nucleolus localization of BLLF2, aa44–48 was therefore another bona fide functional NLS (NLS2). These two functional NLSs are not adjacent to each other, and the functional NLS is generally not long. Thus, the functional NLSs of BLLF2 are aa16–31 and aa44–58. However, no functional NES was identified yet.

**FIGURE 8:**
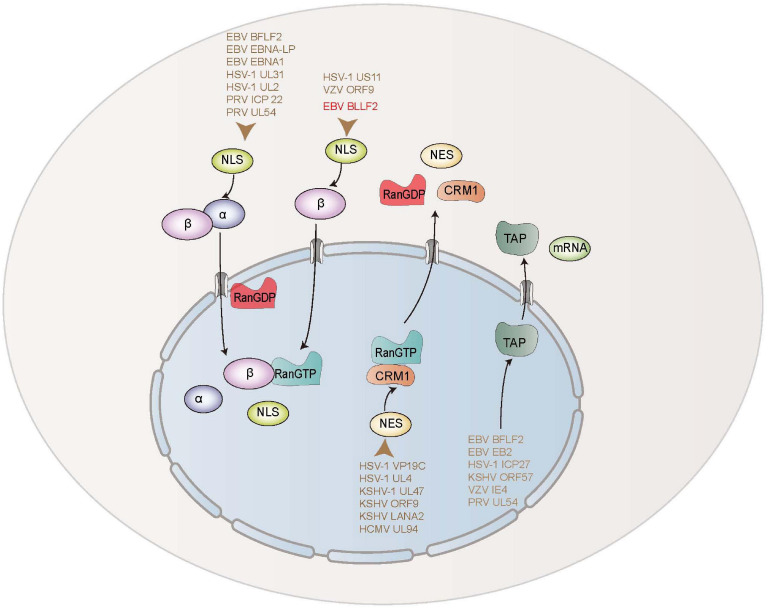
Schematic diagram of nuclear transport mechanisms of EBV and other herpesviruses-encoded proteins.

Nuclear export is a strikingly sophisticated and extremely regulated procedure in cells. The members of the importin/exportin family of nucleocytoplasmic transport receptors, such as TAP and CRM1, are essential for the nuclear export of proteins. For example, the nuclear exports of PRV UL54, EBV BFLF2 and EB2 (Mta) are accomplished by associating with TAP/NXF1 (Buisson et al., 1999; Juillard et al., 2009). HSV-1 ICP27, varicella-zoster virus (VZV) IE4, and Kaposi’s sarcoma-associated herpesvirus (KSHV) ORF57 can bind to several cellular export factors, including SRp20, ASF/SF2, Aly, 9G8, and TAP, to accelerate the export of viral mRNAs *via* the TAP/NXF1 export pathway (Ote et al., 2009; Tunnicliffe et al., 2011). Influenza A virus (IAV) NS1, and herpesvirus saimiri ORF57 are also demonstrated to achieve mRNA export from the nucleus to the cytoplasm by TAP pathway (Fok et al., 2006; Majerciak et al., 2006; Li et al., 2011; Pereira et al., 2017). Additionally, HSV-1 UL4, UL47, and VP19C are manifested to transport to the cytoplasm through functional NES mediated by CRM1-dependent pathway (Williams et al., 2008; Pan et al., 2011; Zhao and Zheng, 2012), which is also exploited by KSHV ORF9, LANA2, and human cytomegalovirus UL94 to fulfill their nuclear exports *via* a classical NES (Munoz-Fontela et al., 2005; Cai et al., 2011; Liu et al., 2012; Figure 8). Besides, the nuclear export activities of chicken anemia virus VP1, IAV NS2, and human immunodeficiency virus type 1 Rev are also modulated *via* a CRM1-mediated pathway (Huang et al., 2013; Behrens et al., 2017; Cheng et al., 2019). In this study, we found that EBV BLLF2 could shuttle between the cytoplasm and nucleus. Although a classical NES in BLLF2 was not identified, we could not rule out that the nuclear export of BLLF2 is mediated through a non-classical pathway or the interaction with other nucleocytoplasmic shuttling proteins, or the spatial constituted functional NES mediates the nucleocytoplasmic shuttling of BLLF2. Accordingly, we continued to identify the nuclear export mechanism of BLLF2 and found that its nuclear export neither depends on CRM1 nor TAP. Therefore, the exact nuclear expert mechanism of BLLF2 needs to be further explored in future studies.

The classical importin-dependent mechanism for nuclear transport is well known for importin α/β/NLS-cargo complex, of which importin α discriminates the NLS, and importin β executes the association with small regulatory Ran-GTP to ship the complex into the nucleus (Wu et al., 2012; Garcia-Yague et al., 2013; Kawashima et al., 2013; Chang et al., 2015; Christie et al., 2016). Moreover, proteins imported into the nucleus can also directly attach to importin β beyond the engagement of importin α-like adaptor (Burton et al., 2017). Up to now, some herpesvirus-encoded proteins are reported to be transported into the nucleus by diverse mechanisms. The nuclear translocation of EBV BFLF2 is mediated through a Ran-, importin α7-, importin β1-, and transportin-1-dependent pathway. EBV EBNA-LP interacts with importin α1 (Nakada and Matsuura, 2017), and EBNA1 binds importin α1 and importin α5 (Nakada et al., 2017) to complete their nuclear traffickings. HSV-1 UL31 is imported into the nucleus through a Ran-, importin α1-, and transportin-1-mediated pathway (Cai et al., 2016b). HSV-1 UL2 is also described to be assisted into the nucleus through Ran, importin α1, α5, α7, β1, and transportin-1 cellular transport receptors (Cai et al., 2017a). PRV ICP22 is demonstrated to be targeted to the nucleus *via* a Ran-, importin α1-, and α7-mediated pathway (Cai et al., 2016a). PRV UL54 is proved to accumulate in the nucleus through a classic Ran-, importin β1-, and importin α5-dependent mechanism (Li et al., 2011). In addition, VZV ORF9 and HSV-1 US11 are shown to be transported into the nucleus *via* a Ran- and importin β-dependent pathway (Xing et al., 2010; Cai et al., 2011; Figure 8). Here, our data disclosed that the nuclear trafficking of BLLF2 was restrained by the Ran-GTP Q69L mutant, indicating that BLLF2 is a Ran-dependent protein. Furthermore, co-transfection of DNs, inhibitors, or shRNA of importins and Co-IP demonstrated that the nuclear import of BLLF2 is mediated by importin β1.

## Conclusion

In conclusion, we identified that EBV BLLF2 is located in the nucleus and nucleolus, which were achieved by two functional NLSs, ^16^KRQALETVPHPQNRGR^31^ (NLS1) and ^44^RRPRPPVAKRRRFPR^58^ (NLS2), and the nucleolus localization signal ^44^RRPR^47^. Additionally, BLLF2 was demonstrated to traffic from the cytoplasm to the nucleus *via* a Ran- and importin β1-dependent mechanism, without importin α. However, the nuclear expert of BLLF2 was mediated neither by a CRM1- nor a TAP-dependent pathway.

## Data Availability Statement

The original contributions presented in the study are included in the article. Further inquiries can be directed to the corresponding author/s.

## Author Contributions

MSC and MLL conceived and designed the experiments. JJL, YJG, YXD, LH, BLL, SYD, JYZ, LX, SXS, XJH, and XLZ performed the experiments and analyzed the data. JJL, MSC, and MLL wrote the manuscript. All authors contributed to and have approved the final manuscript.

## Conflict of Interest

The authors declare that the research was conducted in the absence of any commercial or financial relationships that could be construed as a potential conflict of interest.
